# Effects of Vacuum Freeze-Drying and Spray-Drying Coupled with Maltodextrin and *β*-Cyclodextrin on Cactus (*Opuntia ficus*-*indica*) Postbiotic Powders

**DOI:** 10.3390/foods15162890

**Published:** 2026-08-18

**Authors:** Yuhui Ren, Siyu Ren, Jiahe Li, Weipeng Cui, Yuan Xu, Guangyuan Hua, Changjian Li, Ying Lyu, Hui Xue

**Affiliations:** 1 Key Laboratory of Food Nutrition and Functional Food of Hainan Province, School of Food Science and Engineering, Hainan University, Haikou 570228, China; yuhuiren1210@163.com (Y.R.); rsy914111@163.com (S.R.); lijiahe03346@163.com (J.L.); cwpeng121996@163.com (W.C.); 18995636618@163.com (Y.X.); 2 Hainan State Farms Mushan Coffee Co., Ltd., Qiongzhong 572929, China; huaguangyuan199@yeah.net (G.H.); lichangjian2020@126.com (C.L.); 3 Collaborative Innovation Center of One Health, Hainan University, Haikou 570228, China

**Keywords:** cactus, postbiotic, vacuum freeze-drying, spray-drying, *β*-cyclodextrin

## Abstract

Fruit- and vegetable-derived postbiotic powders represent promising functional ingredients; however, systematic evaluations of different drying technologies and drying aids for these products remain limited. In this study, *Opuntia ficus-indica* fruit juice was fermented using *Lacticaseibacillus paracasei* HNU502 and subsequently inactivated to prepare postbiotic broth, which was further processed into powder by vacuum freeze-drying and spray-drying with maltodextrin and *β*-cyclodextrin as drying aids. Results showed that the drying process and carrier formulation jointly influenced the structural, sensory, and antioxidant characteristics of cactus postbiotic powders. Vacuum freeze-drying combined with *β*-cyclodextrin exhibited favorable structural and sensory properties under the tested conditions, whereas spray-drying combined with *β*-cyclodextrin showed favorable antioxidant performance. Additionally, *β*-cyclodextrin-containing formulations showed favorable powder characteristics and functional properties under the tested conditions, although the effects were influenced by both carrier formulation and drying process. Among the tested formulations, vacuum freeze-drying combined with *β*-cyclodextrin exhibited the highest crystallinity (77.4%) and distinct structural features, as characterized by X-ray diffraction (XRD), scanning electron microscopy (SEM), and Fourier-transform infrared spectroscopy (FTIR) analyses. The spray-dried *β*-cyclodextrin formulation achieved the highest sensory score (74.75), while exhibiting high radical scavenging activity. DSC analysis indicated that all formulations maintained thermal stability, with no exothermic degradation observed below 180 °C. These findings suggest that *β*-cyclodextrin-assisted drying strategies can influence the structural and functional characteristics of cactus postbiotic powders and that drying strategy selection should consider specific product quality requirements and processing feasibility.

## 1. Introduction

Probiotics are defined as live microorganisms that, when administered in adequate amounts, confer beneficial health effects on the host [1]. However, the viability and functional activity of probiotics are highly vulnerable to environmental stresses such as temperature, pH, oxygen, and harsh processing and storage conditions, which greatly limit their industrial applications [2]. Postbiotics refer to bioactive compounds produced during probiotic fermentation, inactivated microbial cells, and/or cell components (e.g., peptidoglycans, exopolysaccharides, organic acids, and bacteriocins) that exert positive physiological and regulatory functions without depending on living microorganisms [3]. Postbiotics exhibit several advantages, including high stability during thermal processing, strong resistance to extreme pH and oxygen, excellent safety, clear composition, and controllable quality [4]. Due to these merits, postbiotics have emerged as a cutting-edge research hotspot in functional foods and show great potential for industrialization [5,6,7].

Vacuum freeze-drying is a low-temperature dehydration technology conducted under vacuum, which minimizes thermal exposure and is widely recognized for preserving the structure and functionality of heat-sensitive compounds [8,9]. Spray-drying is also extensively applied in the food industry for producing powders containing thermally sensitive ingredients due to its rapid, continuous, and scalable operation [10,11]. Although spray-drying involves exposure to elevated temperatures, its short residence time and appropriate selection of drying aids can help minimize thermal damage and maintain product quality. Nevertheless, in our preliminary experiments, direct application of these two drying techniques to postbiotic fermentation broth failed to produce qualified powder products; instead, highly viscous, sticky, and wall-deposited fluids were formed, leading to low powder recovery and difficulties in powder collection and processing [12,13]. As an effective strategy, the addition of food-grade drying aids can significantly improve drying efficiency, reduce matrix viscosity, reduce hygroscopicity, promote particle formation, and enhance the stability and processability of powder products [14].

Maltodextrin and *β*-cyclodextrin are safe, widely used polysaccharide-based drying aids [15]. Maltodextrin, an amorphous linear glucan, can efficiently increase solid content, reduce stickiness, and improve powder formation and recovery efficiency [9,16]. *β*-cyclodextrin, a cyclic oligosaccharide with a hydrophobic internal cavity, can potentially interact with bioactive ingredients through its hydrophobic cavity and has been reported to improve the stability of some bioactive compounds and flavor components [17]. These two carriers have been widely investigated as functional drying aids to alleviate sticking and caking problems, improve powder formation, and enhance the stability of bioactive compounds during drying. However, their effects are highly dependent on carrier concentration, drying conditions, and the physicochemical characteristics of the feed material. During drying, carrier materials can increase the glass transition temperature of the feed matrix, reduce stickiness and wall deposition, improve powder recovery, facilitate particle formation, and reduce thermal or oxidative damage to bioactive compounds by providing a protective matrix.

Opuntia fruit is rich in bioactive constituents, including phenolic compounds, flavonoids, betalains, vitamins, minerals, and dietary fiber, which contribute to its antioxidant and health-promoting properties [18]. Major phenolic constituents reported in cactus fruits include quercetin derivatives, kaempferol glycosides, and phenolic acids such as piscidic and ferulic acids. In addition, cactus mucilage, a characteristic polysaccharide-rich component of Opuntia species, mainly contains arabinose-, galactose-, and rhamnose-containing polymers and contributes to the physicochemical functionality of cactus-derived products [19]. As a pillar industry of Hainan’s characteristic tropical agriculture, cactus cultivation has formed a certain scale and economic benefit. However, the current cactus industry is still dominated by fresh consumption, with a low processing ratio, insufficient deep processing, low product added value, and serious resource waste. Therefore, it is urgent to develop high-value processing technologies and innovative products for cactus. *Opuntia ficus-indica* (family Cactaceae) juice contains abundant nutrients and is easily utilized by probiotics, making it a suitable substrate for preparing high-quality postbiotics [20]. Nevertheless, systematic studies on the preparation, drying, and quality control of cactus-derived postbiotic powder remain limited [21,22,23]. In particular, the interplay between drying conditions and carrier selection—and how this interplay affects the physicochemical, structural, and functional properties of the final powder—has not been systematically elucidated. Addressing this gap is critical for guiding the rational design of high-quality postbiotic powders from fruit and vegetable substrates.

In this study, cactus juice was used as the raw material and fermented with *Lacticaseibacillus paracasei* HNU502 (hereafter referred to as HNU502), followed by inactivation to prepare postbiotic broth [20]. HNU502 is a probiotic strain previously isolated and characterized for its ability to alleviate antibiotic-induced gut microbiota dysbiosis by reestablishing microbial balance and restoring gut mucosal integrity. Importantly, recent studies have demonstrated that HNU502 exhibits excellent fermentation performance in cactus-based substrates, effectively modifying the flavor profile and metabolic characteristics of *Opuntia ficus-indica* fruit juice while significantly inhibiting xanthine oxidase activity, a key marker of oxidative stress. These findings indicate that HNU502 is not only capable of vigorous growth in cactus juice but also contributes to the fermentation characteristics of cactus-based substrates, making it a suitable starter culture for producing cactus-derived postbiotics.

Vacuum freeze-drying and spray-drying were compared, with maltodextrin and *β*-cyclodextrin as drying aids [24]. Physicochemical properties, powder recovery, moisture content, antioxidant activity, sensory characteristics, and structural properties were systematically evaluated. Microstructure, functional group characteristics, crystallinity, and thermal properties were characterized by SEM, FTIR, XRD, and DSC. The purpose of this study was to investigate the effects of drying technologies and drying aids on the quality characteristics of cactus postbiotic powders, identify suitable drying strategies under the tested conditions [20], and provide a theoretical basis for the high-value utilization of fruit and vegetable-derived postbiotic products.

## 2. Experimental Method

### 2.1. Experimental Materials

#### 2.1.1. Raw Materials and Microorganism

Edible cactus fruit puree of *Opuntia ficus-indica* was purchased from Hainan Xianyou Food Co., Ltd. (Haikou, China). The cactus fruits were harvested at full maturity, and the purchased material was already processed into puree by the supplier. After purchase, the puree was stored under refrigerated conditions until use. Before fermentation, the initial pH of the cactus puree was measured using a calibrated pH meter and was 3.85. The cactus puree was used as the fermentation substrate after appropriate pretreatment. *Lacticaseibacillus paracasei* HNU502 was obtained from the culture collection of the College of Food Science and Technology, Hainan University.

#### 2.1.2. Drying Aids and Reagents

Maltodextrin (food-grade, DE 10–15) and *β*-cyclodextrin (food-grade) were purchased from Sinopharm Chemical Reagent Co., Ltd. (Shanghai, China). MRS broth, tryptone, yeast extract, glucose, agar, sodium chloride (NaCl), sodium hydroxide (NaOH), hydrochloric acid (HCl), DPPH (2,2-diphenyl-1-picrylhydrazyl), ABTS (2,2′-azino-bis(3-ethylbenzothiazoline-6-sulfonic acid)), potassium dihydrogen phosphate (KH_2_PO_4_), and disodium hydrogen phosphate (Na_2_HPO_4_) were of analytical grade and obtained from Sango Biotech Co., Ltd. (Shanghai, China). Potassium persulfate and anhydrous ethanol were of analytical grade. Sterile physiological saline (0.85% NaCl) was prepared in the laboratory. Deionized water was used throughout.

### 2.2. Experimental Methods

#### 2.2.1. Pre-Treatment of Cactus Juice

Cactus fruit puree was filtered through a 200-mesh sieve to remove insoluble impurities. The initial pH of the cactus juice was 3.85. To provide a more favorable environment for the growth and fermentation performance of *Lacticaseibacillus paracasei* HNU502, the filtered juice was adjusted to pH 6.0 using 1 mol/L NaOH. The pH-adjusted juice was transferred into conical flasks and sterilized at 121 °C for 15 min. After cooling to room temperature, the sterilized cactus juice was stored under aseptic conditions for subsequent fermentation.

#### 2.2.2. Culture of *Lacticaseibacillus paracasei* HNU502 and Growth Curve Determination

The second-generation seed culture of *Lacticaseibacillus paracasei* HNU502 was inoculated into sterilized cactus juice and incubated at 37 °C under static conditions. At 0, 6, 12, 24, 36, and 48 h, 0.1 mL aliquots were aseptically removed and mixed with 0.9 mL sterile physiological saline (0.85% NaCl) by vortexing for 30 s to prepare a 10^−1^ dilution. Serial ten-fold dilutions were then prepared with sterile saline. For the 0 h time point, dilutions of 10^−3^, 10^−4^, and 10^−5^ were used; for the other time points (6, 12, 24, 36, 48 h), dilutions of 10^−4^, 10^−5^, and 10^−6^ were used. From each dilution, 100 μL aliquots were spread onto MRS agar plates. Three parallel plates were prepared per dilution, and three blank controls (sterile saline) were included at each time point. All plates were incubated at 37 °C for 48 h. Colonies were counted using a colony counter (JC-NJ-3, Shanghai Jingchuang Technology Co., Ltd., Shanghai, China), and the total plate count (CFU/mL) was calculated. The growth curve was plotted with time on the x-axis and log(CFU/mL) on the y-axis. The fermentation time corresponding to the maximum viable cell count was selected as the endpoint for postbiotic broth preparation. Based on the growth kinetics of *Lacticaseibacillus paracasei* HNU502 in cactus juice, 36 h was selected as the fermentation endpoint because the viable cell count reached its maximum value at this time point. After fermentation for 36 h, the broth was subjected to thermal inactivation at 121 °C for 15 min to obtain the postbiotic broth. The inactivation treatment was performed according to commonly applied sterilization conditions for food matrices. Complete microbial inactivation was not experimentally verified in this study.

#### 2.2.3. Characterization of Postbiotic Broth Before Drying

After fermentation, the postbiotic broth was characterized before the addition of drying aids. The soluble solids content was determined using a digital refractometer (PAL-1, Atago Co., Ltd., Tokyo, Japan) and the value was 12.8%. The soluble solids content was used as an indicator of the initial feed composition before drying aid incorporation.

#### 2.2.4. Addition of Drying Aids and Pre-Treatment of Feed Solution

Prior to the formal experiments, preliminary trials were performed to determine suitable drying aid levels based on powder formation characteristics and powder recovery. The minimum drying aid levels required for acceptable powder formation were determined under each drying process, and the selected levels represented the lowest amounts for obtaining powders. The addition amount of each drying aid was calculated relative to the mass of the cactus postbiotic broth. Specifically, the carrier-to-broth ratio was expressed as the mass of drying aid divided by the mass of postbiotic broth. Based on preliminary evaluations, maltodextrin and *β*-cyclodextrin were added at ratios of 0.70 and 1.35 g/g broth, respectively, for vacuum freeze-drying. For spray-drying, maltodextrin and *β*-cyclodextrin were added at a ratio of 0.10 g/g broth, respectively. These formulations were selected according to powder formation performance and recovery characteristics and subsequently applied for further characterization.

Powder recovery was calculated according to the following equation:

Recovery (%) = Wp/[(M_0_ × S) + Mc] × 100%,

where Wp is the mass of collected powder (g), M_0_ is the mass of postbiotic broth before carrier addition (g), S is the soluble solids content of the broth (12.8%), and Mc is the mass of added drying aid (g), which was calculated according to the predetermined carrier-to-broth ratio.

The final total solids content of each feed solution after drying aid incorporation was calculated according to the following equation:

Total solids content (%) = [(M_0_ × S) + Mc]/(M_0_ + Mc) × 100%

where M_0_ is the mass of postbiotic broth before carrier addition (g), S is the soluble solids content of the broth (12.8%), and Mc is the mass of added drying aid (g).

The final total solids contents were 48.71% and 62.89% for vacuum freeze-dried formulations containing maltodextrin and *β*-cyclodextrin, respectively, and 20.73% for both spray-dried formulations.

The selected drying aids were added to the postbiotic broth according to the predetermined carrier-to-broth ratio. The mixtures were stirred at 25 °C and 300 rpm for 30 min using a magnetic stirrer to ensure complete dispersion and dissolution of the carriers. The resulting feed solutions were passed through a 200-mesh sieve to remove insoluble particles before drying. The mixing time was selected based on preliminary trials, during which 30 min was sufficient to achieve homogeneous dispersion of the drying aids in the postbiotic broth without visible agglomeration.

#### 2.2.5. Vacuum Freeze-Drying and Spray-Drying Processes

(1) Vacuum freeze-drying: The filtered feed solution was transferred into freeze-drying trays with a thickness of approximately 8 mm and pre-frozen at −40 °C for 4 h. The pre-frozen samples were subsequently dried using an FD-304 freeze dryer (Jintan Medical Instrument Factory, Changzhou, China). The cold trap temperature was maintained at −50 °C, the vacuum pressure was set at 10 Pa, the sublimation drying temperature was 20 °C, and the final drying temperature was 35 °C. The total drying time was 24 h. After drying, the obtained materials were ground and passed through an 80-mesh sieve to obtain freeze-dried cactus postbiotic powder.
(2) The filtered feed solution was dried using a B-290 spray dryer (Büchi Labortechnik AG, Flawil, Switzerland). The inlet air temperature, outlet air temperature, feed rate, and atomization pressure were set at 180 °C, 85 °C, 15 mL/min, and 0.25 MPa, respectively. The resulting powder was collected from the cyclone separator and stored for subsequent analyses.

#### 2.2.6. Determination of Moisture Content

Moisture content was determined using a DHS-20A electronic moisture analyzer (Shanghai Tianmei Balance Instrument Co., Ltd., Shanghai, China). Approximately 3.5 g of each powder sample was accurately weighed and dried at 105 °C in automatic mode until a constant weight was achieved. Three parallel measurements were performed for each sample, and the moisture content was calculated as

Moisture content (%) = [(m_1_ − m_2_)/m_1_] × 100%,

where m_1_ and m_2_ are the sample masses before and after drying, respectively.

#### 2.2.7. Determination of Antioxidant Activity

(1) DPPH radical scavenging activity

DPPH radical scavenging activity was determined according to the method of Brand-Williams et al. [25], with minor modifications. Postbiotic powder was dissolved in deionized water to concentrations of 1, 2, 4, 8, and 16 mg/mL. A 2 mL aliquot of the sample solution was mixed with 2 mL of a 0.1 mmol/L DPPH ethanol solution, vortexed, and incubated in the dark at room temperature for 30 min. Absorbance was measured at 517 nm using a UV-2600 UV–vis spectrophotometer (Shimadzu Corporation, Kyoto, Japan) and recorded as A_sample_. A _blank_ control (2 mL deionized water instead of sample) was recorded as A_blank_ and a reference control (2 mL ethanol instead of DPPH solution) was recorded as A_reference_. The scavenging rate was calculated as DPPH radical scavenging rate (%) = [1 − (A_sample_ − A_reference_)/A_blank_] × 100%.

(2) ABTS radical scavenging activity

ABTS radical scavenging activity was determined according to the method described by Re et al. [26], with minor modifications. ABTS^+^ stock solution was prepared by mixing 7 mmol/L ABTS with an equal volume of 2.45 mmol/L potassium persulfate and reacting in the dark at room temperature for 12–16 h. The stock solution was diluted with anhydrous ethanol to an absorbance of 0.70 ± 0.02 at 734 nm to obtain the ABTS^+^ working solution. A 0.1 mL aliquot of the sample solution (1–16 mg/mL) was mixed with 3.9 mL of ABTS^+^ working solution, vortexed, and incubated in the dark for 6 min. Absorbance was measured at 734 nm (A_sample_). A _blank_ control (0.1 mL deionized water instead of sample) was recorded as A_blank_. The scavenging rate was calculated as (A_blank_ − A_sample_)/A_blank_ × 100%.

#### 2.2.8. Microscopic and Structural Characterization

(1) Scanning electron microscopy (SEM)

A Quattro S environmental scanning electron microscope (Thermo Fisher Scientific, Waltham, MA, USA) equipped with a gold-sputtering coater (SuPro Instruments, Shenzhen, China) was used. The powder sample was evenly sprinkled onto a sample stage and sputter-coated with gold to improve conductivity. The coated sample was placed into the SEM chamber, and images were acquired at an acceleration voltage of 15 kV at magnifications of 500× and 5000×.

(2) Fourier transform infrared spectroscopy (FTIR)

FTIR spectra were recorded using a Nicolet iS50 FTIR spectrometer (Thermo Fisher Scientific, Waltham, MA, USA). The OMNIC software (version 8.2) was launched, and the instrument was allowed to stabilize. A background scan was performed with 32 scans. The sample (1 part) was mixed with dry KBr (100 parts), ground thoroughly, and pressed into a transparent pellet. The pellet was placed in the sample compartment, and the spectrum was collected over the range of 4000–400 cm^−1^, with a resolution of 4 cm^−1^ and 32 scans. Baseline correction and smoothing were applied using the data processing function. After each measurement, the sample holder was cleaned with a soft cloth to avoid cross-contamination.

(3) X-ray diffraction (XRD)

XRD analysis was performed using an Ultima IV X-ray diffractometer (Rigaku Corporation, Tokyo, Japan). The powder sample was spread flat onto a sample holder. The measurement parameters were Cu Kα radiation (λ = 1.5406 Å), tube voltage 40 kV, tube current 40 mA, scanning range 5–80° (2θ), scanning speed 4°/min, and step size 0.02°. The measurement time was approximately 20–30 min per sample. The diffraction pattern was recorded for structural analysis.

(4) Differential scanning calorimetry (DSC)

DSC thermograms were obtained using a DSC 250 differential scanning calorimeter (TA Instruments, New Castle, DE, USA). Approximately 5–10 mg of powder sample was placed in an aluminum pan, and an empty aluminum pan was used as a reference. The temperature range was set from 20 °C to 200 °C with a heating rate of 10 °C/min under a nitrogen atmosphere (flow rate 50 mL/min). The DSC curve was recorded, and the glass transition temperature (Tg) was determined.

#### 2.2.9. Sensory Evaluation

Sensory evaluation was performed using reconstituted cactus postbiotic powder samples to simulate the intended consumption form of the product. For sample preparation, 15 g of each cactus postbiotic powder formulation was accurately weighed and reconstituted with 300 mL of warm deionized water (30 °C). The reconstituted beverages were thoroughly mixed to ensure uniform dispersion and were then equally distributed into 20 sensory cups (approximately 15 mL per cup) for evaluation by the panelists.

A panel of 20 trained food specialists evaluated the reconstituted samples based on odor, color, mouthfeel, consistency, and taste using a 100-point scoring system. In this study, mouthfeel referred to the perceived textural characteristics of the reconstituted product. Higher total scores indicated better sensory acceptability. All sensory evaluations were conducted in a standard sensory laboratory under controlled conditions.

#### 2.2.10. Electronic Tongue Analysis

The taste profiles of cactus postbiotic powders were evaluated using an ASTREE electronic tongue (Metrohm AG, Herisau, Switzerland) equipped with seven potentiometric sensors (AHS, SCS, ANS, CPS, NMS, CTS, PKS) and an Ag/AgCl reference electrode. Data acquisition and analysis were performed using the instrument software (version 7.3.2). For sample preparation, 3.5 g of each powder was placed into a 50 mL centrifuge tube, mixed with 35 mL of ultrapure water, thoroughly vortexed, and then centrifuged at 8000 r/min for 15 min; the supernatant was collected for analysis. Three standard solutions—0.01 mol/L HCl (sourness), 0.01 mol/L NaCl (saltiness), and 0.01 mol/L monosodium glutamate (MSG, umami)—were prepared in 100 mL volumetric flasks for sensor calibration. Prior to sample analysis, the sensors were conditioned and calibrated according to the manufacturer’s instructions. Approximately 30 mL of the supernatant was transferred into a standard beaker, and the sensor array was immersed. Measurements were performed at room temperature with a data acquisition time of 120 s per sample, and the response values (in mV) were recorded for each sensor. Between measurements, the sensors were thoroughly rinsed with ultrapure water until a stable baseline was restored.

### 2.3. Data Processing

All experiments were conducted in parallel three times, and the experimental data were expressed as “mean ± standard deviation”. IBM SPSS Statistics (version 26.0, IBM Corp., Armonk, NY, USA) was used for statistical analysis. Two-way analysis of variance (ANOVA) was performed to evaluate the effects of drying process and drying aid formulation on the measured quality parameters. Because carrier levels differed among formulations, the statistical results were interpreted together with the formulation-dependent experimental design rather than as a fully independent factorial comparison. When a significant interaction was detected, one-way ANOVA followed by Duncan’s multiple range test was used to compare individual group means. A *p*-value < 0.05 was considered statistically significant. OriginPro 2024 (OriginLab Corporation, Northampton, MA, USA) was used for data visualization.

After preparation, all cactus postbiotic powders were immediately transferred into airtight polyethylene bags and stored at 4 °C until further analysis. All physicochemical, structural, antioxidant, and sensory analyses were completed within two weeks after powder preparation. The storage temperature and packaging conditions were maintained consistently for all samples to minimize variations caused by moisture absorption and environmental exposure.

## 3. Results and Discussion

### 3.1. Growth Curve of Lacticaseibacillus paracasei HNU502

**Figure 1:**
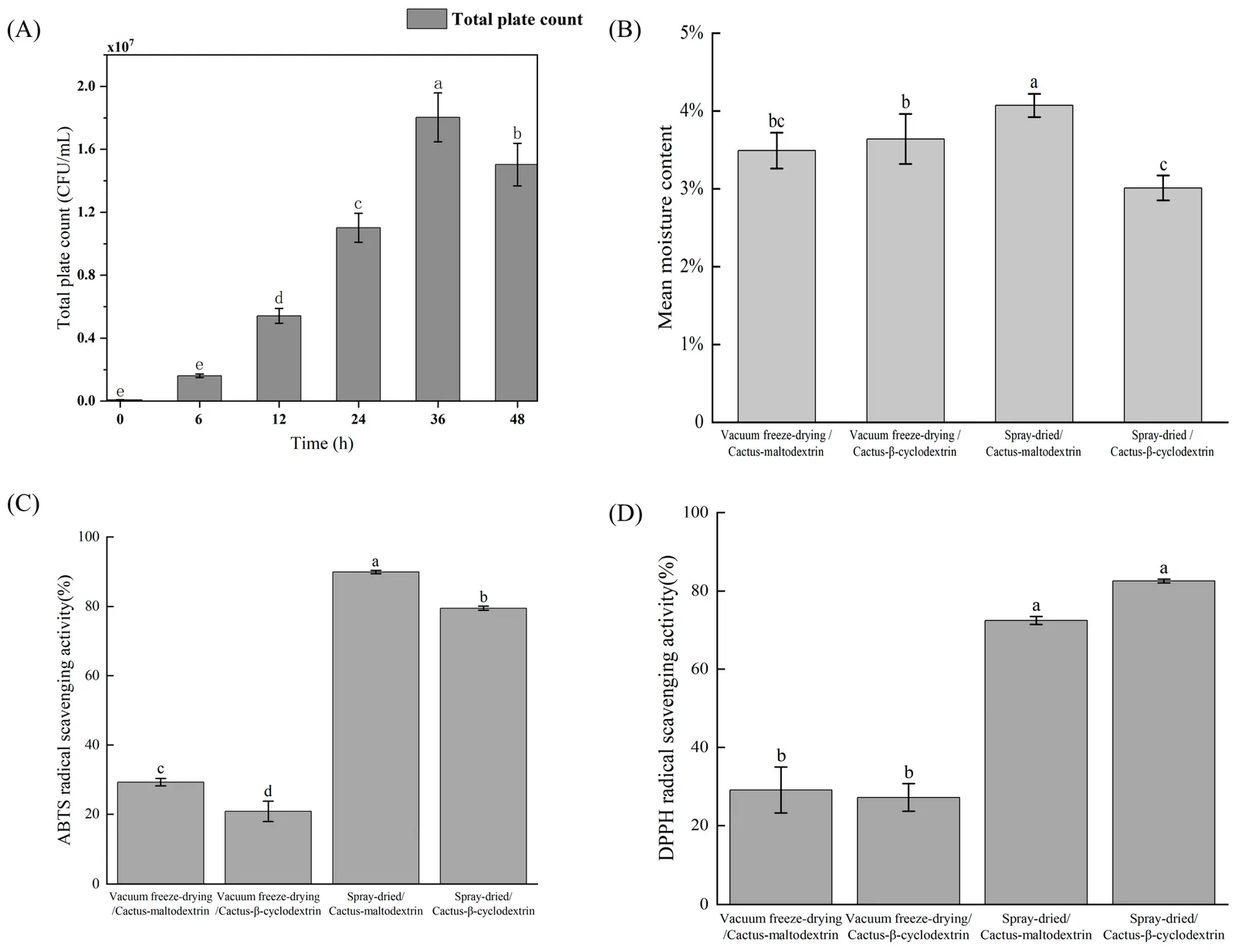
Physicochemical, proliferative, and antioxidant properties of cactus postbiotic powders prepared with maltodextrin and *β*-cyclodextrin by vacuum freeze-drying and spray-drying. (**A**) Total viable plate count of probiotics cultured with cactus postbiotic supernatant at different incubation times. (**B**) Moisture content of four powder formulations (%). (**C**) ABTS radical scavenging inhibition (%). (**D**) DPPH radical scavenging inhibition (%). Values are expressed as mean ± standard error. Different lowercase letters denote significant differences at *p* < 0.05.

The growth profile of HNU502 in cactus juice followed a typical microbial proliferation pattern (Figure 1A). After a short lag phase (0–6 h), the strain entered the exponential growth phase, during which cell density increased rapidly. The maximum total plate count reached 1.8 × 10^7^ CFU/mL at 36 h, followed by a slight decline to 1.5 × 10^7^ CFU/mL at 48 h, indicating entry into the stationary and early decline phases. The synchronous decrease in pH confirmed active organic acid production. These results verify that cactus juice provides a favorable environment for HNU502 growth, laying a foundation for subsequent postbiotic preparation.

### 3.2. Powder Recovery of Cactus Postbiotic Powders

Powder recovery was evaluated to assess the efficiency of powder collection under different drying processes and carrier formulations. The recovery values were calculated based on the collected powder mass relative to the calculated solid input, including the original solids present in the postbiotic broth and the added drying aids. The final feed compositions differed among formulations due to the different carrier addition levels applied under each drying condition. The vacuum freeze-dried formulations showed relatively high powder recovery rates, reaching 87% and 86% for maltodextrin- and *β*-cyclodextrin-containing powders, respectively. In comparison, the spray-dried formulations exhibited lower powder recovery rates of 26% and 37% for maltodextrin- and *β*-cyclodextrin-containing powders, respectively. The relatively lower recovery observed during spray-drying was mainly attributed to the limited sample quantity used in the laboratory-scale experiments and unavoidable material loss during atomization and collection. These results indicate that powder recovery was influenced by the combined effects of drying process, carrier formulation, and feed composition under the tested conditions.

### 3.3. Moisture Content of Cactus Postbiotic Powders

Moisture content is an essential index for evaluating the quality of powdered food products, which closely correlates with physical stability, hygroscopicity, and microbial safety of powders [27,28,29]. In this study, cactus postbiotic powders were prepared using maltodextrin (MD) and *β*-cyclodextrin (*β*-CD) as drying aids across all experimental groups [30].

For samples produced by vacuum freeze-drying, the moisture content of the powder incorporated with maltodextrin was 3.49%, while that of the *β*-cyclodextrin group was 3.64%. Both values remained below 4%, which is generally considered favorable for maintaining powder physical quality [29] and reducing microbial deterioration in dehydrated food powders. Statistical analysis further verified that no significant difference existed between the two freeze-dried groups. It can be concluded that under vacuum freeze-drying conditions, the variation of drying aids barely alters the residual moisture of the obtained powders [31,32].

Differing from the freeze-drying results, the selection of drying aids exerted a remarkable effect on moisture content during spray-drying. The powder with maltodextrin had a moisture content of 4.07%, whereas the corresponding value of the *β*-cyclodextrin group decreased substantially to 3.01%, showing a statistically significant difference between the two formulations. The lower moisture content observed in the *β*-CD spray-dried powder may be attributed to the cyclic molecular structure of *β*-cyclodextrin, which exhibits lower water-binding capacity and facilitates moisture removal during atomization and drying. In contrast, maltodextrin possesses numerous hydroxyl groups and a highly amorphous structure, which tends to retain a greater amount of residual water after drying. Similar phenomena have been reported in spray-dried fruit and plant-derived powders formulated with cyclodextrin-based carriers [24].

Overall, the moisture level of cactus postbiotic powders varied considerably with drying methods and drying aids. These results indicate that moisture characteristics were influenced by the combined effects of drying process and carrier composition. Since different carrier levels were applied for the two drying technologies, the observed differences should be interpreted as formulation-dependent rather than solely attributed to the drying method itself.

In the present study, moisture content was selected as an indicator of dehydration performance and residual water level in the dried matrix. All cactus postbiotic powders exhibited low moisture contents (<4%), indicating effective water removal during the drying processes. Low moisture levels may reduce the availability of water within the powder matrix and are generally associated with lower risks of moisture-related deterioration. However, moisture content alone does not directly represent water activity and cannot fully predict powder stability, including microbial growth, caking behavior, or storage performance. Therefore, the moisture content results in this study provide information regarding dehydration characteristics and powder quality under the tested conditions rather than direct evidence of long-term storage stability. The evaluation of powder quality was based on moisture content together with physicochemical, structural, antioxidant, and sensory characteristics. The low moisture contents observed in all formulations indicate favorable dehydration performance and may reduce the potential risks associated with moisture-related deterioration and microbial proliferation under the tested conditions.

### 3.4. Antioxidant Activity of Cactus Postbiotic Powders

The antioxidant activities of cactus postbiotic powders were evaluated using ABTS and DPPH radical scavenging assays (Figure 1C,D). Significant differences were observed among the four formulations, indicating that both drying conditions and carrier composition influenced the measured antioxidant activity of the resulting powders. However, these differences should be interpreted with consideration of the dilution effects caused by the incorporation of drying aids, as carrier levels varied among the tested formulations.

For ABTS radical scavenging activity, the spray-dried maltodextrin sample showed the highest value (0.8987 ± 0.005), followed by the spray-dried *β*-cyclodextrin sample (0.7949 ± 0.006). Notably, the spray-dried *β*-cyclodextrin sample achieved significantly higher measured activity than both vacuum freeze-dried formulations (*p* < 0.05). This result suggests that *β*-cyclodextrin-containing formulations may provide a favorable matrix for maintaining antioxidant activity during spray drying; however, the contribution of carrier composition and concentration to the measured values cannot be excluded.

For DPPH radical scavenging activity, the spray-dried *β*-cyclodextrin sample recorded the highest value (0.8257 ± 0.0046), which was significantly higher than the vacuum freeze-dried samples (*p* < 0.05) and comparable to the spray-dried maltodextrin sample (0.7247 ± 0.0103, *p* > 0.05). This indicates that *β*-cyclodextrin-containing spray-dried powders exhibited higher DPPH radical scavenging activity under the tested conditions. The observed activity differences may be associated with differences in carrier composition and matrix characteristics.

Across both assays, spray-dried powders showed higher measured antioxidant activity than freeze-dried powders under the tested formulations. These results suggest that the combination of spray-drying and *β*-cyclodextrin may contribute to favorable antioxidant performance; however, the observed differences reflect the combined effects of drying conditions and carrier formulation rather than drying technology alone. Critically, the spray-dried *β*-cyclodextrin sample exhibited the most consistent and robust antioxidant performance, ranking top in DPPH activity and maintaining strong ABTS activity. This balanced performance indicates that *β*-cyclodextrin-containing formulations exhibited favorable antioxidant characteristics under spray-drying conditions. However, the measured antioxidant activity may also be influenced by carrier composition, carrier level, and matrix effects.

### 3.5. Microstructural Characterization of Cactus Postbiotic Powders

**Figure 2:**
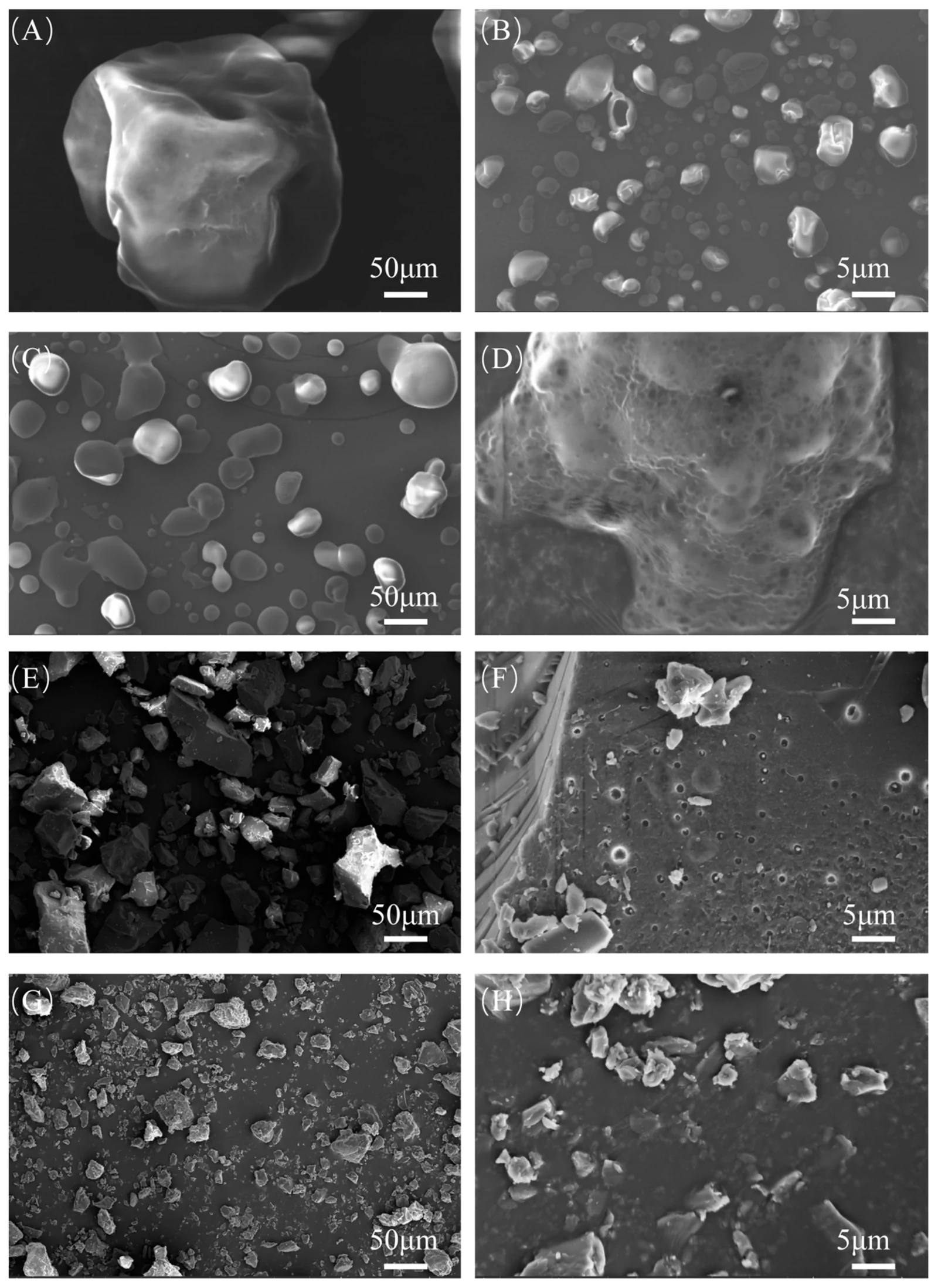
Scanning electron micrographs of cactus postbiotic powders prepared by spray-drying (**A**–**D**) and vacuum freeze-drying (**E**–**H**), with maltodextrin (**A**,**B**,**E**,**F**) or *β*-cyclodextrin (**C**,**D**,**G**,**H**) as drying aids. Images (**A**,**C**,**E**,**G**) were captured at 500× magnification; images (**B**,**D**,**F**,**H**) were captured at 5000× magnification.

Figure 2 presents the SEM micrographs of cactus postbiotic powders prepared using vacuum freeze-drying and spray-drying with maltodextrin or *β*-cyclodextrin as drying aids. Distinct differences in particle morphology and surface structure were observed among the four formulations, suggesting that the resulting microstructure was influenced by the combined effects of the drying process, carrier composition, and post-drying processing procedures [24].

The spray-dried powders (Figure 2A–D) exhibited relatively discrete and particle-like structures with smaller particle sizes, which is consistent with the typical particle formation mechanism of atomization-based drying. In contrast, the vacuum freeze-dried samples (Figure 2E–H) displayed larger irregular fragments with porous and rough surfaces, which can be attributed to the formation of a porous frozen matrix during sublimation followed by mechanical grinding after drying. The maltodextrin-containing spray-dried sample (Figure 2A,B) showed relatively spherical particles with smooth surfaces, whereas the *β*-cyclodextrin-containing spray-dried sample (Figure 2C,D) exhibited more irregular particle surfaces and increased aggregation. These differences suggest that carrier composition influenced particle formation behavior during spray-drying. Based on SEM observations, spray-dried particles were generally within the micrometer range and exhibited relatively smaller and more discrete structures, whereas freeze-dried powders consisted of larger irregular fragments due to grinding after sublimation.

The vacuum freeze-dried powders (Figure 2E–H) displayed larger irregular fragments and porous surface structures. The maltodextrin-containing freeze-dried sample (Figure 2E,F) showed fractured structures with relatively compact regions, whereas the *β*-cyclodextrin-containing freeze-dried sample (Figure 2G,H) exhibited more fragmented and porous features with visible cavities and rough surfaces. These characteristics are associated with ice crystal sublimation during freeze-drying, which generates a porous dried matrix, followed by the mechanical grinding process required to convert the dried material into powder.

Overall, the SEM results demonstrated that the morphology of cactus postbiotic powders was influenced by both the drying process and drying aid selection. Spray-drying produced relatively discrete particles due to direct droplet atomization and rapid moisture removal, whereas vacuum freeze-drying resulted in porous and irregular fragments formed after ice crystal sublimation and subsequent mechanical size reduction. In addition, differences between maltodextrin- and *β*-cyclodextrin-containing formulations indicate that carrier composition also contributed to the final powder microstructure.

### 3.6. Chemical Structure (FTIR) Analysis

**Figure 3:**
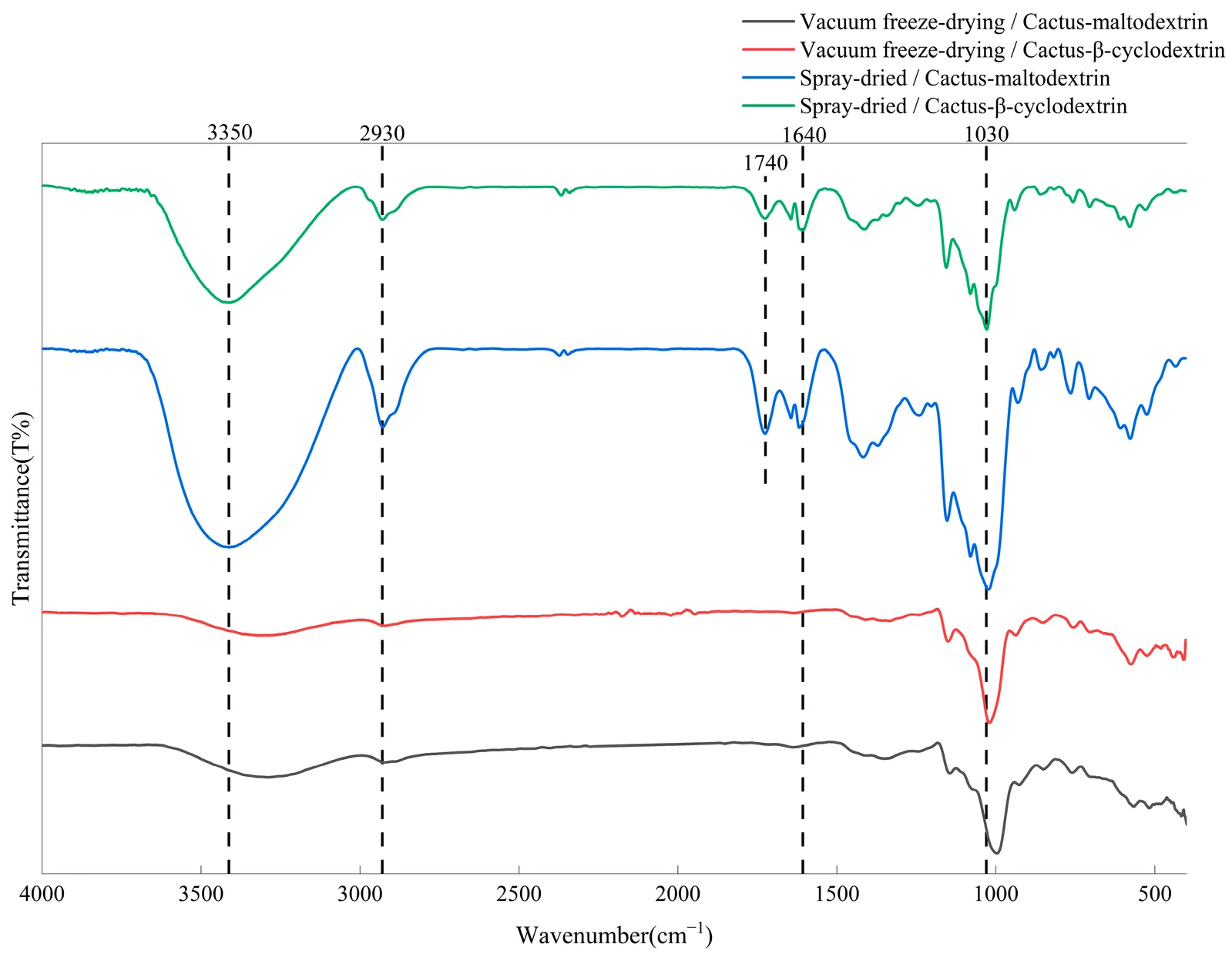
Fourier-transform infrared spectra of cactus postbiotic powders supplemented with *β*-cyclodextrin or maltodextrin via vacuum freeze-drying and spray-drying. Key characteristic bands are indicated at 3350, 2930, 1740, 1640, and 1030 cm^−1^.

The functional group characteristics of cactus postbiotic powders were characterized by FTIR spectroscopy, as shown in Figure 3. All four formulations (vacuum freeze-dried cactus–*β*-cyclodextrin, vacuum freeze-dried cactus–maltodextrin, spray-dried cactus–*β*-cyclodextrin, and spray-dried cactus–maltodextrin) exhibited typical polysaccharide-related absorption patterns, indicating that the main chemical functional groups of the cactus postbiotic powders were maintained after drying.

Across all samples, key characteristic bands were consistently observed at ~3350 cm^−1^ (O–H stretching vibration, indicative of hydroxyl groups and hydrogen bonding), ~2930 cm^−1^ (C–H asymmetric stretching vibration of aliphatic chains), ~1740 cm^−1^ (C=O stretching vibration, associated with carbonyl groups), ~1640 cm^−1^ (C=C stretching/amide I band, related to unsaturated bonds or protein structures), and ~1030 cm^−1^ (C–O–C stretching vibration of glycosidic bonds). These signals confirm the presence of polysaccharide, protein, and hydroxyl-containing components in all powders, consistent with the expected composition of cactus postbiotic products.

A clear effect of the drying aid was evident in both processes. Differences in FTIR profiles were observed between *β*-cyclodextrin- and maltodextrin-containing formulations, particularly in the hydroxyl (3350 cm^−1^) and glycosidic (1030 cm^−1^) regions. This pattern suggests that *β*-cyclodextrin-containing formulations exhibited different spectral characteristics and matrix organization compared with maltodextrin-based formulations, which may be associated with differences in carrier structure. In contrast, maltodextrin-based samples displayed broader, less defined absorption bands, consistent with the linear amorphous structure of maltodextrin, which primarily functions as a physical carrier within the powder matrix.

Notably, the vacuum freeze-dried cactus–*β*-cyclodextrin powder exhibited the most distinct and well-resolved peaks across all key regions. The higher intensity and sharper signals observed at 3350 and 1030 cm^−1^ suggest differences in molecular organization and functional group environments within the powder matrix, likely facilitated by the low-temperature, slow dehydration conditions of freeze-drying. These observations suggest that freeze-drying combined with *β*-cyclodextrin resulted in a different molecular arrangement within the powder matrix.

Spray-dried samples showed slightly broader peaks than freeze-dried ones, possibly due to minor structural relaxation induced by thermal and shear stresses during atomization and drying [33].

However, no significant shifts or disappearances of characteristic bands were observed, confirming that spray drying also preserved the main functional groups. Even under spray-drying conditions, the *β*-cyclodextrin-containing sample maintained better-defined peaks than the maltodextrin formulation, indicating that *β*-cyclodextrin-containing formulations maintained different molecular characteristics under spray-drying conditions.

Collectively, these FTIR results demonstrate that both drying methods maintained the characteristic functional groups of cactus postbiotic powders. The differences observed between *β*-cyclodextrin- and maltodextrin-containing formulations suggest that carrier type influenced the molecular organization of the powder matrix. However, the observed spectral differences mainly reflect variations in functional group environments and matrix organization among formulations. These findings suggest that *β*-cyclodextrin was associated with differences in the molecular organization of cactus postbiotic powders.

### 3.7. X-Ray Diffraction (XRD) Analysis

The crystallinity and structural characteristics of cactus postbiotic powders were influenced by both the drying process and the choice of drying aid. Among the tested formulations, *β*-cyclodextrin-containing powders exhibited distinct crystallization behaviors under different drying conditions, suggesting that carrier structure and processing environment jointly contributed to the final crystalline properties.

**Figure 4:**
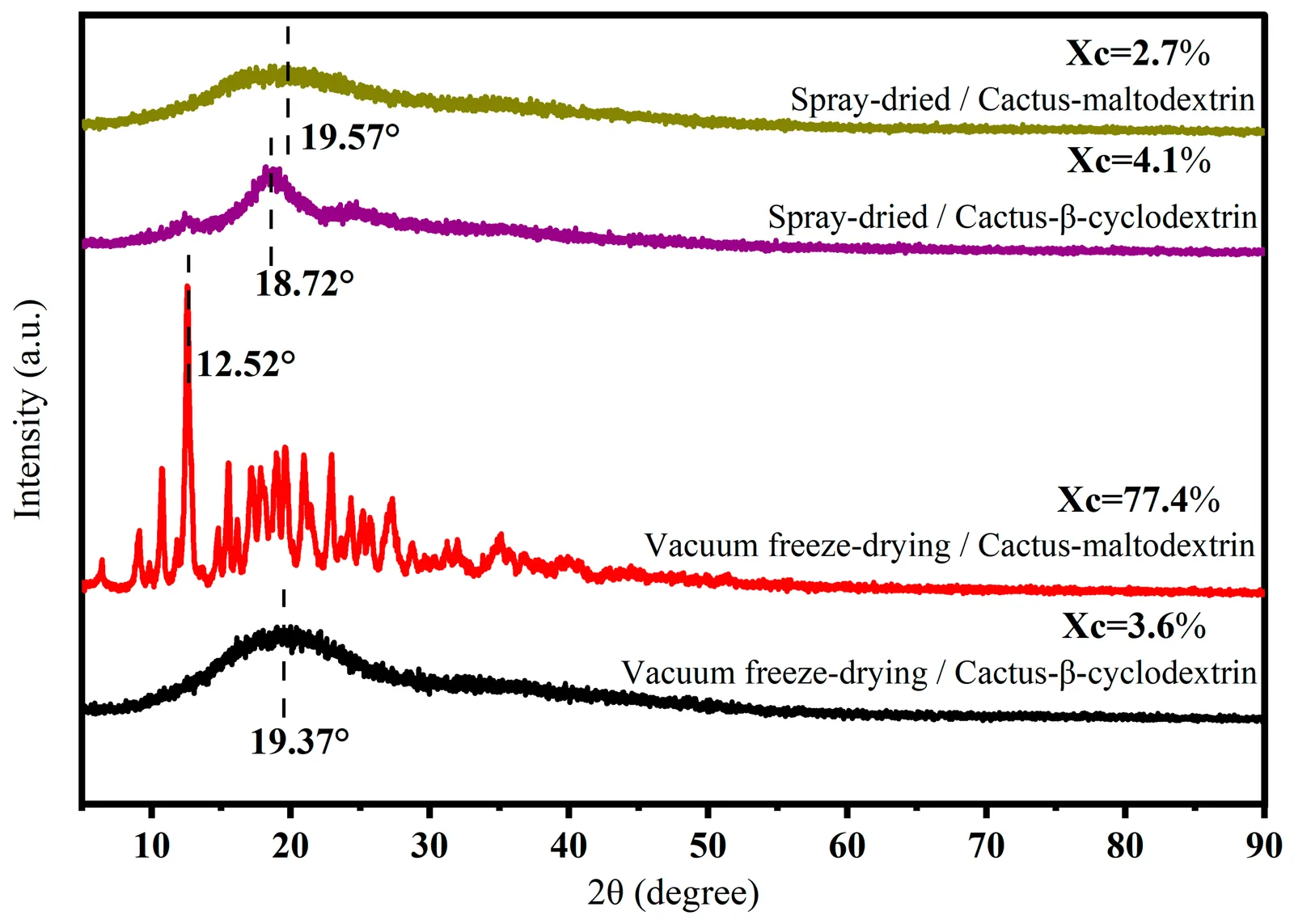
X-ray diffraction patterns and relative crystallinity of cactus postbiotic powders obtained by different drying processes.

In the vacuum freeze-dried samples, *β*-cyclodextrin incorporation resulted in a well-defined crystalline structure, evidenced by multiple sharp, intense diffraction peaks (Figure 4). The primary diffraction peak was observed at 2θ = 12.52°, and the crystallinity (Xc) reached 77.4%, far exceeding all other formulations. The high crystallinity observed in the vacuum freeze-dried *β*-cyclodextrin formulation may be associated with the slow dehydration characteristics of freeze-drying, which allow greater molecular rearrangement during ice sublimation. In addition, the molecular structure and higher carrier level of *β*-cyclodextrin in this formulation may have contributed to the formation of ordered crystalline regions through intermolecular interactions. Such a highly crystalline structure is generally associated with improved thermal and physical stability in dried powder systems, which may be advantageous for long-term storage. In contrast, the vacuum freeze-dried powder formulated with maltodextrin exhibited only a broad, diffuse halo centered at 2θ = 19.37°, with a very low crystallinity of 3.6%. This behavior reflects the inherent amorphous nature of maltodextrin, whose randomly arranged short molecular chains cannot form long-range ordered structures even under conditions favorable for crystallization, resulting in a predominantly amorphous matrix [34].

For the spray-dried formulations, the effect of the drying aid on crystallinity was markedly different. The spray-dried *β*-cyclodextrin-containing powder showed only a broad, low-intensity peak centered at 2θ = 18.72°, with no sharp crystalline reflections and a crystallinity of only 4.1%. Similarly, the spray-dried maltodextrin-based powder displayed a diffuse halo at 2θ = 19.57° and a crystallinity of just 2.7%, consistent with its inherent amorphous character. The rapid dehydration conditions during spray-drying may restrict molecular rearrangement and reduce the formation of highly ordered crystalline structures. However, the difference between freeze-dried and spray-dried samples should be interpreted cautiously because the two drying processes were conducted with different carrier levels, which may also influence crystallization behavior.

Collectively, these results indicate that the crystalline characteristics of cactus postbiotic powders were influenced by the combined effects of drying process and carrier composition. Vacuum freeze-drying combined with *β*-cyclodextrin exhibited the highest crystallinity among the tested formulations, which may be related to both the slow dehydration mechanism and the structural characteristics of the carrier. However, because carrier levels differed between drying treatments, the contribution of carrier concentration to crystallization behavior cannot be excluded. Therefore, the observed crystallinity differences should be interpreted as the combined effects of drying process, carrier formulation, and carrier level rather than the independent effect of drying technology.

### 3.8. Thermal Properties Characterized by DSC

**Figure 5:**
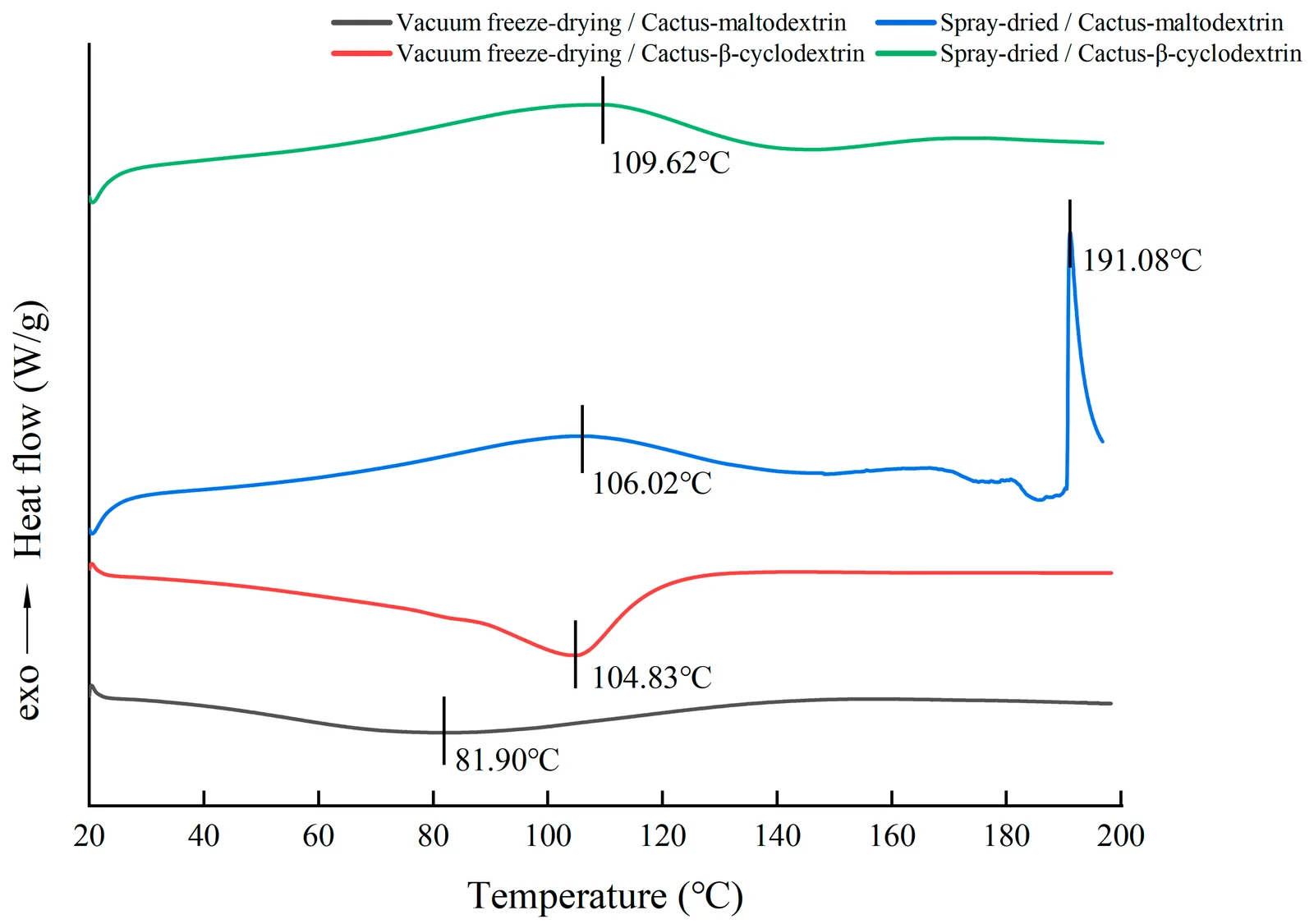
DSC thermograms of cactus postbiotic powders with different drying aids and drying processes. All curves are vertically offset for clearer visual comparison; exothermic direction points upward. Heating rate: 10 °C/min from 20 to 200 °C.

Differential scanning calorimetry (DSC) was employed to evaluate the thermal behavior of cactus postbiotic powders produced via vacuum freeze-drying and spray-drying, using maltodextrin and *β*-cyclodextrin as drying aids. The corresponding thermograms are presented in Figure 5.

All samples exhibited a broad endothermic event spanning 40–120 °C, which arises from the combined effects of free and bound water evaporation, as well as the glass transition of the amorphous powder matrix.

For the vacuum freeze-dried samples, the cactus-maltodextrin formulation displayed a mild, broad endothermic hump centered at 81.90 °C, with a gradual transition profile. This observation suggests differences in matrix organization and water-associated thermal transitions between formulations. These characteristics may be related to the low-temperature dehydration process of freeze-drying as well as the molecular properties of the carrier materials. In contrast, the vacuum freeze-dried cactus-*β*-cyclodextrin sample showed a pronounced water-loss endothermic valley at 104.83 °C, reflecting the combined effects of residual water desorption and glass transition in the amorphous matrix [35].

Among the spray-dried samples, the cactus–maltodextrin formulation exhibited a broad endothermic transition peaking at 106.02 °C, followed by a sharp, intense endothermic melting peak at 191.08 °C. This high-temperature peak is tentatively attributed to the melting of thermally stable crystalline regions within the spray-dried maltodextrin matrix. However, the specific molecular origin of this transition cannot be determined solely from the DSC profile. The spray-dried cactus-*β*–cyclodextrin sample demonstrated the flattest baseline and weakest endothermic fluctuation across 40–120 °C, with only a shallow, broad endothermic hump at 109.62 °C. This thermal profile suggests that *β*-cyclodextrin-containing formulations exhibited different water-associated transitions and matrix responses during spray-drying. The observed differences may be related to the molecular structure of *β*-cyclodextrin and its interaction with other components in the powder matrix; however, the influence of carrier level differences among formulations should also be considered.

Notably, no exothermic degradation peaks were observed for any sample below 180 °C, indicating that all tested formulations maintained thermal stability within the evaluated temperature range. Collectively, the DSC results suggest that *β*-cyclodextrin influenced the thermal behavior of cactus postbiotic powders through interactions with the powder matrix. However, the effects of carrier composition, carrier concentration, and drying conditions cannot be completely separated in the current experimental design.

### 3.9. Sensory Evaluation of Cactus Postbiotic Powders

**Figure 6:**
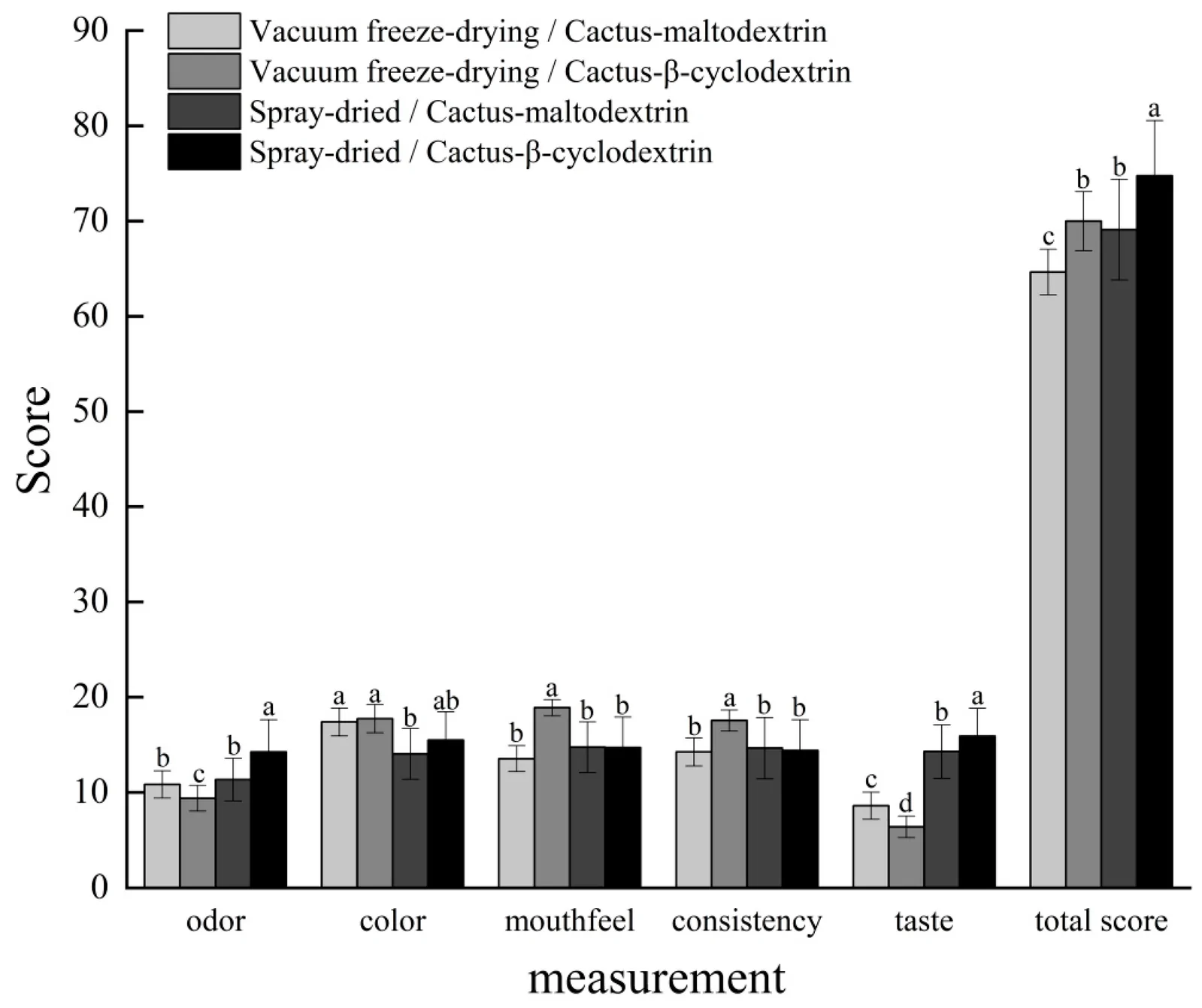
Sensory profiles of reconstituted cactus postbiotic powder samples produced with different drying aids and drying processes. Values are presented as mean ± standard error. Different lowercase letters denote significant differences (*p* < 0.05) within the same sensory attribute.

The sensory quality of reconstituted cactus postbiotic powder samples prepared via vacuum freeze-drying and spray-drying, using maltodextrin and *β*-cyclodextrin as drying aids, was evaluated across five sensory attributes: odor, color, mouthfeel, consistency, and taste. The total score was used to represent the overall sensory acceptability of the reconstituted products (Figure 6). Significant differences in sensory profiles were observed among the four formulations, driven by both the drying process and the choice of drying aid.

For vacuum freeze-dried samples, the formulation containing *β*-cyclodextrin exhibited a significantly higher total sensory score (70.00 ± 3.11) than the maltodextrin-containing counterpart (64.65 ± 2.39, *p* < 0.05). Specifically, the *β*-cyclodextrin sample showed higher mouthfeel (18.90 ± 0.85 vs. 13.55 ± 1.36, *p* < 0.05) and consistency (17.55 ± 1.10 vs. 14.25 ± 1.48, *p* < 0.05) scores, which may be related to its more ordered crystalline microstructure, as observed by XRD. Notably, its odor score was slightly lower (9.40 ± 1.35 vs. 10.85 ± 1.42, *p* < 0.05), which may be attributed to differences in flavor release behavior after reconstitution; yet, this effect did not compromise the overall sensory acceptability. Color scores remained comparable between the two freeze-dried formulations (17.75 ± 1.48 vs. 17.40 ± 1.46, *p* > 0.05), indicating that both drying aids effectively preserved the natural color of the cactus matrix during freeze-drying.

For spray-dried samples, the *β*-cyclodextrin-containing powder achieved the highest total sensory score (74.75 ± 5.81), significantly outperforming the maltodextrin formulation (69.10 ± 5.29, *p* < 0.05). Notably, the spray-dried *β*-cyclodextrin sample scored significantly higher for odor (14.25 ± 3.38 vs. 11.35 ± 2.25, *p* < 0.05) and taste (15.90 ± 2.94 vs. 14.30 ± 2.81, *p* < 0.05), which may be related to the molecular structure of *β*-cyclodextrin and its potential influence on flavor characteristics after reconstitution and can also be associated with differences in carrier composition and matrix characteristics after reconstitution. While color, mouthfeel, and consistency scores were similar between the two spray-dried formulations (*p* > 0.05), the improvement in key flavor attributes contributed to higher overall sensory performance.

Across both drying processes, *β*-cyclodextrin-containing formulations showed favorable sensory characteristics under the tested conditions. In contrast, maltodextrin showed limited efficacy in improving sensory attributes, particularly flavor-related scores, due to its weaker ability to interact with volatile compounds and modulate powder texture. These findings indicate that *β*-cyclodextrin-containing formulations showed favorable sensory characteristics under the tested conditions.

### 3.10. Electronic Tongue Taste Profiles

**Figure 7:**
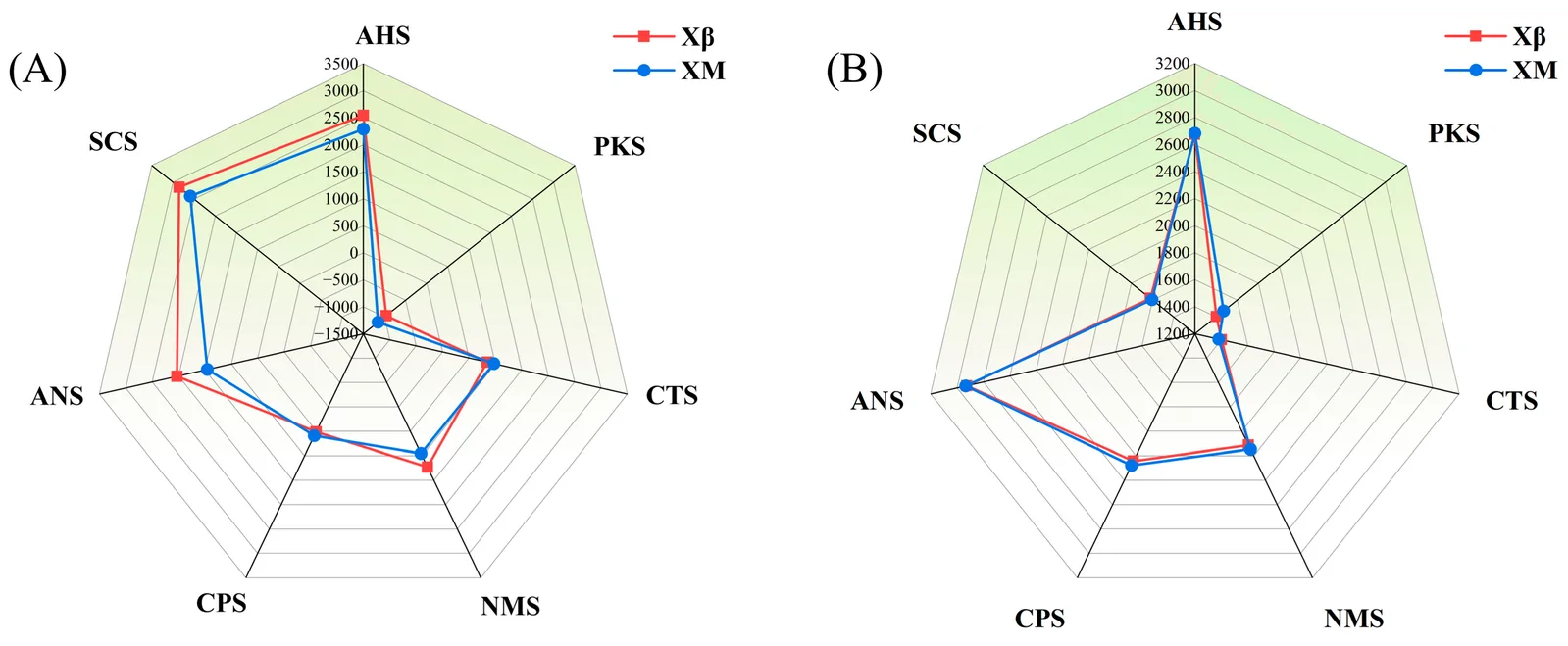
Electronic tongue radar plots of cactus postbiotic composite powders. (**A**) Vacuum freeze-dried samples. (**B**) Spray-dried samples. AHS, sourness; SCS, sweetness; ANS, umami; CPS, bitterness; NMS, saltiness; CTS, aftertaste-astringency; PKS, aftertaste-bitter.

The taste profiles of cactus postbiotic powders were evaluated using an electronic tongue equipped with seven sensors (AHS: sourness, SCS: sweetness, ANS: umami, CPS: bitterness, NMS: saltiness, CTS: aftertaste-astringency, PKS: aftertaste-bitter). Figure 7 shows the radar plots for samples containing *β*-cyclodextrin (X*β*) or maltodextrin (XM) under vacuum freeze-drying (A) and spray-drying (B).

Vacuum freeze-drying is shown in Figure 7A. Both X*β* and XM exhibited the highest response in AHS, indicating that sourness was the dominant taste characteristic, largely unaffected by the drying aid. Compared with XM, X*β* gave significantly higher responses in AHS, SCS, and ANS, corresponding to stronger overall taste intensity, particularly enhanced sweetness and umami. XM showed a milder and more harmonious but weaker profile, with no significant differences in CTS and NMS between the two formulations. These results suggest that *β*-cyclodextrin-containing formulations showed enhanced taste responses in freeze-dried samples.

Spray-drying is shown in Figure 7B. X*β* displayed moderate and uniform responses across all seven indices, with AHS and SCS at relatively high levels and ANS, CPS, and NMS at medium intensity. In contrast, XM showed an uneven distribution: the highest response was in ANS (umami), and its AHS value was slightly higher than that of X*β*. SCS and CPS responses were similar between the two samples, but XM exhibited lower NMS (saltiness), CTS, and PKS (aftertaste) values, indicating a reduction in these taste attributes. Thus, *β*-cyclodextrin preserved the intrinsic core taste and maintained a balanced flavor profile during spray-drying, whereas maltodextrin altered the overall fingerprint by enhancing umami but suppressing saltiness and aftertaste.

Overall, the electronic tongue analysis indicated that *β*-cyclodextrin-containing formulations exhibited distinct taste profiles under both drying processes. These differences suggest that carrier composition influenced the taste characteristics of cactus postbiotic powders; however, the overall sensory effects may also depend on the interactions between carrier type and drying conditions [36].

## 4. Discussion

In this study, the effects of vacuum freeze-drying and spray-drying, combined with maltodextrin (MD) or *β*-cyclodextrin (*β*-CD) as drying aids, on the physicochemical, structural, antioxidant, and sensory properties of cactus postbiotic powders were systematically investigated. The results demonstrated that both drying process and carrier type influenced the characteristics of the resulting powders. Vacuum freeze-drying combined with *β*-cyclodextrin showed favorable structural characteristics and sensory quality under the tested formulation, whereas spray-drying combined with *β*-cyclodextrin exhibited favorable antioxidant performance. These findings indicate that the suitability of a drying strategy depends on the specific quality attributes considered rather than a single universally superior process.

The favorable structural characteristics observed in vacuum freeze-dried powders can be associated with the low-temperature dehydration process, which minimizes thermal exposure and facilitates porous structure formation. In contrast, spray-drying involves instantaneous high-temperature exposure, which tends to promote bioactive degradation, browning reactions, and volatile aroma loss, ultimately compromising product quality. These findings are consistent with previously reported comparisons of drying technologies for postbiotic and bioactive powder preparation [37].

The distinct effects of the two drying aids on powder quality can be explained by their molecular structures and interaction mechanisms. Maltodextrin, as an amorphous linear polysaccharide, increases the solid content and reduces the viscosity of the feed solution, thereby facilitating droplet formation during spray-drying and ice crystal growth during freeze-drying [24]. Its linear chains physically disrupt the continuous postbiotic matrix, creating irregular, blocky particles with improved flowability and dispersibility [16,38]. However, maltodextrin lacks specific molecular binding sites, so its protection of bioactive compounds is limited to physical dilution and matrix modification [39].

In contrast, *β*-cyclodextrin possesses a cyclic oligosaccharide structure with a hydrophobic inner cavity, which has been reported to interact with some non-polar bioactive molecules through inclusion phenomena [40,41]. While direct evidence of inclusion complex formation (e.g., from NMR or ITC) was not obtained in this study, several observations suggest possible differences in matrix organization. First, the FTIR spectra of *β*-CD-containing powders exhibited differences in peak intensity and profile compared with MD-based formulations, suggesting differences in molecular environment and matrix organization. Second, the higher crystallinity observed by XRD (77.4% for freeze-dried *β*-CD vs. 3.6% for freeze-dried MD) indicates that *β*-CD promotes the formation of an ordered crystalline matrix, consistent with its known tendency toward crystallization. Third, the endothermic melting peak observed at 191.08 °C in the spray-dried maltodextrin formulation indicates the presence of thermally stable structures within the powder matrix. This thermal transition may be associated with the thermal behavior of the maltodextrin-containing matrix and the rearrangement of components during drying. The interpretation of this transition should be considered together with the results obtained from FTIR and XRD analyses, which provide complementary information regarding functional groups and structural organization. Collectively, the structural characteristics observed by FTIR, XRD, and DSC may contribute to differences in antioxidant performance among formulations. Nevertheless, because carrier levels differed among treatments, the effects of carrier-related dilution, matrix composition, and drying conditions should be considered when interpreting antioxidant activity. Previous studies have reported that cyclodextrins combined with polysaccharide-based wall materials can provide complementary functions, in which cyclodextrins may contribute to molecular interactions while polysaccharide carriers improve powder formation and processability. Such synergistic effects may partially explain the favorable functional properties observed in *β*-CD-containing formulations. Moreover, *β*-CD-containing formulations showed distinct surface characteristics in SEM analysis. However, the observed porous and fragmented structures were also influenced by the drying mechanism, particularly the sublimation process during vacuum freeze-drying and subsequent powder processing.

The differences observed in FTIR profiles among formulations suggest variations in molecular environment and matrix organization. The higher crystallinity observed in vacuum freeze-dried *β*-CD samples indicates that the drying process and carrier composition jointly influenced structural rearrangement. DSC results further suggested differences in thermal transitions among formulations. However, these structural analyses do not provide direct evidence of specific inclusion complexes or molecular interactions, which require further chemical characterization.

Two-way ANOVA revealed significant interactions between drying method and carrier type for several quality parameters (*p* < 0.05), indicating that the effectiveness of a given carrier is dependent on the drying technology employed. This underscores the importance of optimizing carrier selection in conjunction with the drying process rather than treating them as independent variables.

The levels of drying aids were determined based on preliminary trials aimed at identifying the minimum addition levels required to achieve acceptable powder formation under each drying process. Therefore, different carrier-to-broth ratios were applied for vacuum freeze-drying and spray-drying. This strategy was designed to investigate the performance of cactus postbiotic powders prepared with the lowest feasible amount of drying aid under each processing condition. Consequently, the observed differences among formulations should be interpreted as the combined effects of the drying process, carrier formulation, and carrier level rather than the independent effect of drying technology alone.

The comprehensive analysis of physicochemical, structural, antioxidant, and sensory indicators identified vacuum freeze-drying combined with *β*-cyclodextrin as a formulation with favorable structural and sensory characteristics under the tested conditions. These findings provide a theoretical basis and practical guidance for the development of high-value functional foods from cactus and other fruit- and vegetable-derived postbiotic products. Nevertheless, the industrial adoption of vacuum freeze-drying must consider its high energy consumption, batch-wise operation, and significant capital costs, which may limit its scalability for large-volume production compared with the more continuous and cost-effective spray-drying process.

**Figure 8:**
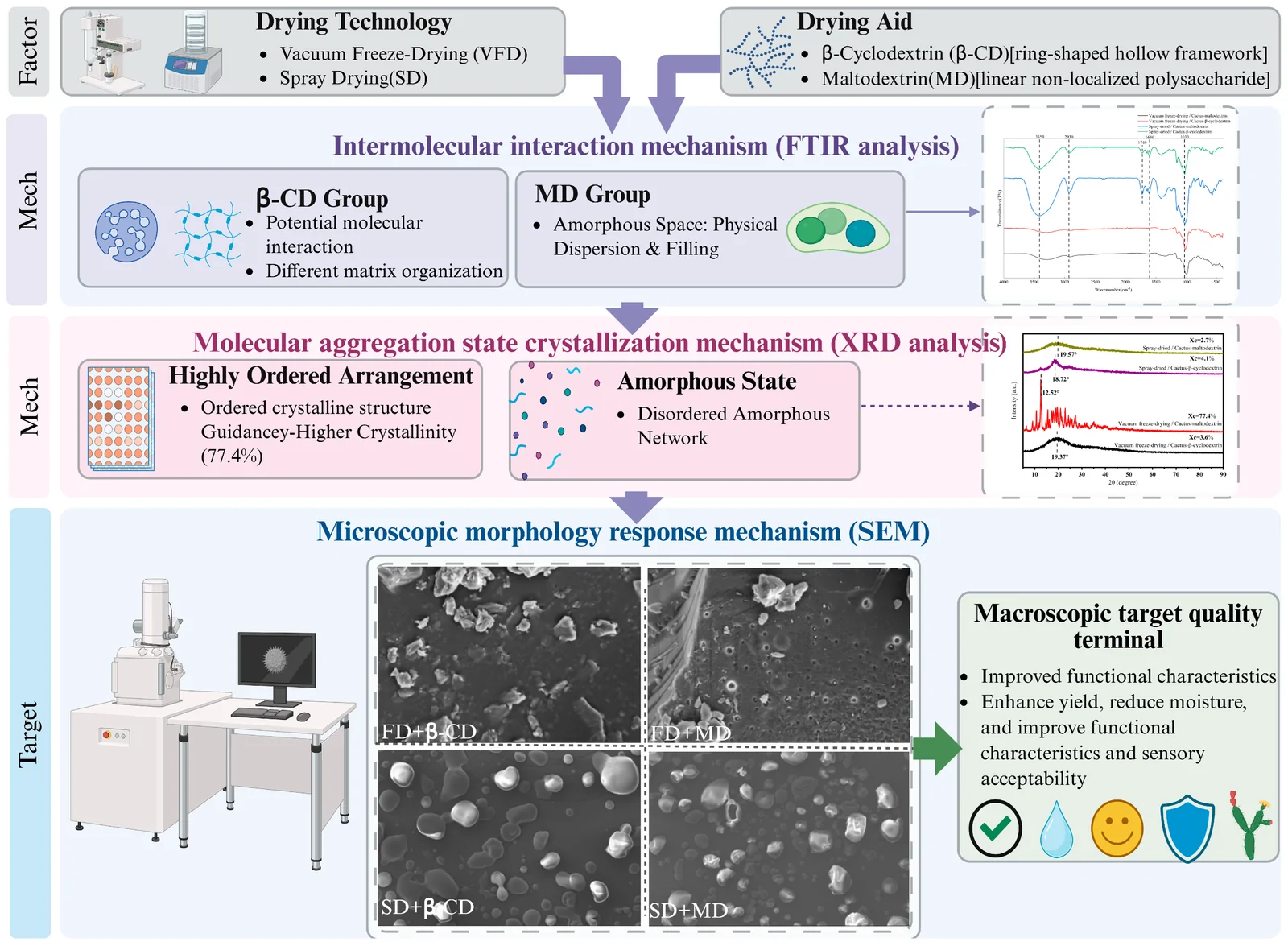
Multi-scale mechanistic framework of cactus postbiotic powder formation regulated by drying technologies and drying aids, spanning molecular organization, crystallinity evolution, microstructural changes, and macroscopic quality outcomes.

The antioxidant evaluation in this study was based on overall radical scavenging capacity rather than the identification or quantification of individual postbiotic components. Therefore, the observed differences in antioxidant performance should be interpreted as the combined effects of drying conditions, carrier formulation, and matrix characteristics rather than direct evidence of changes in specific fermentation-derived metabolites or bioactive compounds. The combination of antioxidant assays, structural characterization, and physicochemical analyses provided a comprehensive evaluation of powder functional characteristics under the investigated conditions. The relationships among drying conditions, carrier characteristics, matrix organization, microstructure, and powder quality are summarized in the conceptual framework shown in Figure 8.

## 5. Conclusions

In this study, cactus juice was fermented with *Lacticaseibacillus paracasei* HNU502 and subsequently inactivated to produce postbiotic broth, which was then converted into powder using vacuum freeze-drying and spray-drying with maltodextrin or *β*-cyclodextrin as drying aids. The results demonstrated that both the drying technology and carrier type significantly influenced the physicochemical properties, antioxidant activity, sensory quality, and structural characteristics of the resulting powders. Under the tested formulations, vacuum freeze-drying combined with *β*-cyclodextrin showed favorable structural characteristics and sensory quality, whereas spray-drying combined with *β*-cyclodextrin exhibited favorable antioxidant performance. These differences should be interpreted considering the combined effects of drying conditions and carrier formulation. The combined analysis of physicochemical, structural, and sensory data indicated that the vacuum freeze-dried *β*-cyclodextrin formulation showed favorable structural characteristics among the tested conditions. This observation may be associated with its higher crystallinity and distinct matrix organization; however, the contribution of carrier concentration and drying conditions cannot be excluded. However, it is important to recognize that vacuum freeze-drying is an energy-intensive batch process with high operational costs, which may limit its direct scalability for large-scale industrial production. Conversely, spray-drying remains a more cost-effective and continuous alternative, although different quality attributes should be considered when selecting the drying strategy. Therefore, the selection of an appropriate drying strategy should be guided by a balanced consideration of product quality requirements, production scale, and economic feasibility. Nonetheless, our findings provide useful insights into the development of cactus-derived postbiotic powders and suggest that *β*-cyclodextrin showed potential as a drying aid under the investigated conditions.

## Data Availability

The original contributions presented in this study are included in the article. Further inquiries can be directed to the corresponding author.
